# Aggravation from Jarring

**Published:** 1895-04

**Authors:** E. V. Ross

**Affiliations:** Rochester, N. Y.


### AGGRAVATION FROM JARRING.

E. V. Ross, M. D., Rochester, N. Y.

In the February issue of THE HOMŒOPATHIC PHYSICIAN the editor calls attention to a characteristic of Belladonna, viz.: "Aggravation from jarring of the bed." I have so frequently verified this indication that I desire to add my mite of testimony. Only recently I met with a case where this modality was present. Bell.$^{40m}$ brought relief within five minutes from the intense agonizing pains from which the patient was suffering. Another characteristic of this remedy and one of equal value is: "Pains come and go suddenly." When these two characteristics are present at the same time, it is needless to look any further for a remedy.

We believe the symptom should read, "*Aggravation from jarring,*" for it matters not whether the patient be in bed or sitting in a chair, so long as the symptoms are aggravated from jarring. Referring to Guernsey's *Key-Notes to Mat. Med.*, p. 73, we find: "*Worse from the least jar of the bed or chair, which aggravates the patient exceedingly.*"

For the past four years I have been collecting material which I intend (when the time and spirit moves me) to report, to show the efficacy of Homœopathy in pain in opposition to the prevailing belief among many so-called homœopaths that in order to "hold your patients" you must use the "squirt-gun method." On reviewing the material at hand I have been impressed with the frequency which these so called "key-note" or characteristic symptoms appear. I am not in favor of "key-note" prescribing, but at times they are of inestimable value in helping one out in the hour of need, and when you are at a loss to find a remedy covering the symptom totality (*Simillimum*). And here let me say it is well for one to be grounded in the *Organon;* but he should know something of the *Materia Medica* and not be content with carrying it around in his "grip," but carry a certain amount of it in his head—at least a few of the *characteristics.*

